# Differences of Clonogenic Mesenchymal Stem Cells on Immunomodulation of Lymphocyte Subsets

**DOI:** 10.1155/2018/7232717

**Published:** 2018-09-09

**Authors:** Pascual Martínez-Peinado, Sandra Pascual-García, Enrique Roche, José Miguel Sempere-Ortells

**Affiliations:** ^1^Immunology Division, Biotechnology Department, University of Alicante, San Vicente del Raspeig, Alicante, Spain; ^2^Biochemistry and Cell Therapy Unit, Institute of Bioengineering, University Miguel Hernandez, Elche, Alicante, Spain

## Abstract

Mesenchymal stem cells (MSC) are a widely used population in cell therapy for their ability to differentiate into distinct tissues and more lately, for their immunomodulatory properties. However, the use of heterogeneous populations could be responsible for the nondesired outcomes reflected in the literature. Here, we analyse the different capacities of five one-cell-derived MSC clones to exert their immunomodulation ex vivo. We assessed proliferation assays in cocultures of MSC clones and purified cluster of differentiation (CD)3^+^, CD4^+^, or CD8^+^ lymphocytes; analysed the regulatory T (Treg) cells fold change rate; determined the effects on viability of peripheral blood mononuclear cells (PBMC); and also measured the coculture cytokine profiles (Th1/Th2). Conditioned media (CM) of different clones were also used to perform both proliferation assays and to analyse Treg fold change. The five clones analysed in this work were able to generate heterogeneous environments. Different clones inhibited proliferation of CD3^+^ and CD4^+^ lymphocytes, with different intensities. Surprisingly, all clones promoted proliferation of CD8^+^ lymphocytes. Different MSC clones and their CM were able to increase the number of Treg with different intensities. Finally, different clones also promoted different effects on the viability of PBMC treated with ultraviolet light. Considering all these data together, it seems that different clones, even from the same donor, can promote a wide spectrum of responses from anti-inflammatory to proinflammatory character. This fact may be important to standardise the design of personalized cell therapy protocols, thus diminishing the aforementioned undesired outcomes existing nowadays in this type of therapies.

## 1. Introduction

Mesenchymal stem cells (MSC) are stem cells that can be isolated from tissues of adult organisms. They were discovered by Friedenstein et al. [1–3] in the late 70's in the bone marrow of mice and guinea pigs, and since then, they have been isolated from numerous tissues, such as the umbilical cord [4], dental pulp [5], and adipose tissue [6, 7], among many others. These MSC are a cell type with great potential for cell therapy, as well as for the treatment of autoimmune/autoinflammatory diseases [8]. This potential lies in the possibility of isolating them from the adult organism, diminishing their ethical implications; in their ability to differentiate into osteogenic [9], adipogenic [10], and chondrogenic [11] lineages; in the possibility to be transdifferentiated into other cell types, such as neurons [12]; in their medium-low expression of major histocompatibility complex (MHC) class I and MHC class II [13], which allows their use in allogeneic therapies [14], and finally, in their immunomodulatory properties, which promote, among other responses, an inhibition of most immune cell types function [15], as well as an increase in the number and activity of regulatory T cells (Treg) [16].

The mechanisms by which MSCs exert their immunomodulatory effects involve a multitude of soluble factors [17] and cell-to-cell contact [18], although the degree of contribution of each of these factors in such immunomodulation remains a matter of debate nowadays. Moreover, this immunomodulation has been studied mostly on total PBMCs, with only a few studies carried out on specific lymphocyte populations, such as CD3, CD4, or CD8 lymphocytes. In addition, the heterogeneity of MSC [19], their multiple origins, the differences in isolation methods, and the absence of a single marker that allows us to correctly identify them, may be ultimately responsible for the wide range of published outcomes [20, 21].

In our previous work [22], we used clonal populations of MSC, derived from adipose tissue, previously isolated using cloning rings [23], in order to homogenize the population as much as possible. In that work, we demonstrated the different capacities of MSC clones to exert immunosuppression on total PBMC populations; secrete different cytokines with or without stimulation; and show different intensities and percentages of expression of the markers generally used to identify them, like cluster of differentiation (CD)44, CD73, CD90, and CD105, and different gene methylation profiles related to cytokine signalling of each one of the clones.

In this work, we delve deeper into the study of these clones, analysing their effect on purified populations of T lymphocytes, the cytokine environment resulting from cocultivation with PBMC, the ability of clones to modify the Treg population, the effect of CM on PBMC and Treg proliferation, and finally, the effect of these clones on the viability of PBMC exposed to proapoptotic stimuli.

## 2. Materials and Methods

### 2.1. Cells and Reagents

All procedures involving human cells were approved by the University of Alicante Ethics Committee. PBMC were obtained by centrifugation in the density gradient in Ficoll-Hypaque (GE Healthcare, Chalfont, St Giles, UK) from the antecubital vein of 57 healthy volunteers. Total T lymphocytes, as well as T helper (Th) and T cytotoxic (Tc) cell subpopulations, were purified by incubating the PBMC with the RosetteSep Human T Cell Enrichment Cocktail, RosetteSep Human CD4^+^ T Cell Enrichment Cocktail, and RosetteSep Human CD8^+^ T Cell Enrichment Cocktail (StemCell Technologies, Vancouver, BC, Canada), respectively, prior to gradient density centrifugation with Ficoll-Hypaque. For PBMC culture, complete RPMI (Roswell Park Memorial Institute) media was used to grow PBMC, consisting in RPMI (Hyclone, Logan, UT, USA) supplemented with 10% fetal bovine serum (FBS) (Biowest, Nuaillé, France), 1% penicillin/streptomycin (Biowest, Nuaillé, France), and 1% glutamine (Biowest, Nuaillé, France).

Clones were isolated from heterogeneous MSC populations derived from liposuction of two healthy volunteers, as previously described [23], three of them from one individual (clones 1.10, 1.22, and 1.7) and two of them from the other one (clones 3.10 and 3.5). Briefly, heterogeneous MSC populations were cultivated in plates at very low confluence to identify individual cells in complete cloning medium [HAM F-12 (Gibco, Carlsbad, CA, USA) supplemented with 20% FBS, 100 U/mL penicillin, 100 *μ*g/mL streptomycin, and 15 mM HEPES (Gibco, Carlsbad, CA, USA)] until defined colonies (30–50 cells) were formed. Once the colonies were identified, they were isolated using cloning rings to limit their confluence. Then, the clones were detached using trypsin 0.05% with ethylenediaminetetraacetic acid (EDTA) 0.02% (Gibco, Paisley, UK) and transferred to new culture flasks in complete Dulbecco's Modified Eagle's Medium (DMEM), consisting in DMEM 25 mM glucose (Gibco, Paisley, UK), supplemented with 10% FBS, 1% penicillin/streptomycin, and 1% glutamine. Membrane antigen expressions (CD44, CD73, CD90, and CD105) were confirmed by flow cytometry [22]. All the clones used in this work were between passages 6–10 for proliferation assays, 10–12 for cytokine's profiles, 11–14 for Treg analysis, and 12–15 for the viability assay. MSC clones were grown in complete DMEM. Media were renewed twice a week until cell confluence was reached. Afterwards, media were washed twice with phosphate-buffered saline (PBS) (Hyclone, Logan, UT, USA), and cells were detached from the flask with trypsin to carry out the different experiments or to make further passages in order to continue expanding.

Monoclonal antibodies for CD4, CD25, and Forkhead Box P3 (FoxP3) antigens for flow cytometry were purchased from eBioscience (San Diego, CA, USA) and CD11b from Becton Dickinson (San Diego, CA, USA). Fixation/permeability solution for intranuclear staining of FoxP3 staining was purchased from eBioscience (San Diego, CA, USA). Carboxyfluorescein succinimidyl ester (CFSE) and phytohaemagglutinin (PHA) were purchased from Sigma-Aldrich (Saint-Quentin Fallavier, France) and propidium iodide from Fisher Scientific (Leicestershire, UK).

### 2.2. MSC Conditioned Media

To obtain MSC conditioned media (CM), MSC clones were tripsinized and seeded in a 25 cm^2^ flask (TPP, Trasadingen, Switzerland) at the rate of 10^5^ cells per flask, in 4 mL of complete DMEM. After 96 hours of culture, supernatants were collected and centrifuged at 360g for 5 minutes. Then, they were frozen at −20°C until their usage.

### 2.3. Analysis of Cytokines in MSC-PBMC Cocultures

Cytokine analysis in coculture supernatants was performed by seeding 10^5^ PBMC in 96 flat-bottom well plates (TPP, Trasadingen, Switzerland), with and without the different MSC clones at 1 : 10 ratio (MSC : PBMC), in complete RPMI medium for 96 hours. They were stimulated with 10 *μ*g/mL of PHA, and no stimulation condition was used as control. Supernatants were then collected, centrifuged at 360g, and frozen at −20°C until analysis. Cytokine detection was performed by flow cytometry (FacsCanto II, Becton Dickinson, San Diego, CA, USA) and the Human Th1/Th2/Th9/Th17/Th22 13plex FlowCytomix Multiplex kit from eBioscience (San Diego, CA, USA). The analysed cytokines were interleukin- (IL-) 1*β*, tumour necrosis factor- (TNF-) *α*, IL-10, interferon- (IFN-) *γ*, IL-4, IL-2, IL-22, IL-13, IL-17A, IL-9, IL-5, and IL-12p70. Data were analysed using FlowCytomix Pro Software (eBioscience, San Diego, CA, USA).

### 2.4. CFSE Labelling

To analyse cell proliferation, prior to culture, PBMC and purified CD3^+^, CD4^+^, or CD8^+^ lymphocytes were labelled with CFSE at a final concentration of 5 *μ*M, for 5 min at room temperature, according to Parish and Warren [24].

### 2.5. Proliferation Assay

To analyse the effect of MSC-CM on PBMC proliferation, 10^5^ cells were cultured in 96 flat-bottom well plates in a final volume of 200 *μ*L consisting in 100 *μ*L of complete RPMI with 100 *μ*L of every MSC-CM. 100 *μ*L of complete DMEM was used as the control. To analyse the effect of MSC clones on proliferation of purified CD3^+^, CD4^+^, and CD8^+^ lymphocyte subsets, 10^5^ lymphocytes were cultured with MSC clones (ratio MSC : PBMC, 1 : 10) in 96 well plates in a final volume of 200 *μ*L of complete RPMI. All the cultures were stimulated with 10 *μ*g/mL of PHA. Nonstimuli condition was used as the control in all cases. After five days of culture, cells were collected, and proliferation was measured by flow cytometry (EPICS-XL, Beckman Coulter, Brea, CA).

### 2.6. Treg Analysis

Analysis of the effect of MSC on the Treg cells was performed by seeding 10^5^ PBMC with the different MSC clones at an MSC : PBMC ratio (1:10) in 200 *μ*L complete RPMI medium, on 96-well flat-bottom plates. The condition without MSC was used as the control. Similarly, the effect of MSC-CM was also analysed by seeding 10^5^ PBMC in 100 *μ*L of complete RPMI media and 100 *μ*L of CM from each one of the clones. Complete DMEM was used as the negative control. After three days of culture, the cells were recovered and stained with monoclonal antibodies against the membrane antigens CD4 and CD25, as well as the intranuclear transcription factor FoxP3. Cells were processed by flow cytometry (EPICS-XL, Beckman Coulter, Brea, CA).

### 2.7. Viability Assay

To assess the effect of MSC clones on PBMC survival, 10^5^ PBMC were cocultured with clones 1.10 and 3.5 (ratio MSC : PBMC, 1 : 20), in a 96-well flat-bottom plate, at a final volume of 200 *μ*L of complete RPMI. After 6 days of coculture, cells were moved to a new 96-well flat-bottom plate and then irradiated with ultraviolet (UV) light through a transilluminator for 30 seconds. Twenty-four hours later, they were stained with propidium iodide (10 *μ*g/mL) and analysed by flow cytometry (EPICS-XL, Coulter). Condition without UV stimulation was used as a basal mortality control.

### 2.8. Statistics

Descriptive statistics and statistical inference were performed using the GraphPad Prism (GraphPad, La Jolla, CA, USA) software statistical analysis package. In all cases, the results were checked for normality by the Kolmogorov-Smirnov test. Later, Student's *t*-test for two sets of data was used to analyse significant differences between the different growing conditions. *F* test of the analysis of variance (ANOVA) was used to analyse clones among themselves, or between themselves and the control condition. Post hoc analysis was performed with Fisher's least significant difference (LSD) method.

## 3. Results

### 3.1. Th1/Th2/Th17 Profile in MSC Clones-PBMC Cocultures

#### 3.1.1. IL-1*β*

Under nonstimulatory conditions (Table 1), the presence of MSC clones resulted in an increase in IL-1*β* levels. This increase was statistically significant for all clones, with no differences between them, except for pair 1.7 and 1.22, with the higher and the lower increase, respectively. PHA stimulation (Table 2) led to an increase in this cytokine in all cases, being statistically significant for clones 1.7 and 3.10. The rest of clones did not substantially modify IL-1*β* levels with respect to the condition of absence of MSC clones.

#### 3.1.2. IL-2

This cytokine showed a variable behaviour. Its expression was either absent or very low (close to 50 pg/mL). Although no significant differences were found between the different growing conditions or between clones, it could be observed that, in the presence of clones in the culture, there was generally less cytokine production, both with (Table 2) or without PHA (Table 1). There was an undetectable amount of cytokine in the cases of clones 1.10, 1.7, and 3.5 (in the absence of stimuli) and 3.5 and 3.10 (in the presence of stimuli).

#### 3.1.3. IL-12p70

Only the presence of clone 1.7 and with PHA stimulation (Table 2) led to detection of this cytokine at a very low concentration (about 40 pg/mL).

#### 3.1.4. IL-17A

No cytokine was detected in any of the growing conditions without stimuli (Table 1). However, in the presence of PHA (Table 2), moderate levels of IL-17 were detected in the supernatants of PBMC alone. This amount doubled in the presence of the MSC clones, except for clone 1.10, which showed a contrary trend to the other ones, since the concentration of this cytokine decreased significantly.

#### 3.1.5. IL-22

In the absence of stimulus (Table 1), only very low levels of IL-22 (about 50 pg/mL) were detected in the presence of clone 1.10. However, PHA stimulation (Table 2) was associated with low-moderate levels of this cytokine in PBMC alone (about 150 pg/mL). This amount decreased to a lesser or greater extent depending on the MSC clone present in the culture and even became undetectable in the case of clones 1.10 and 3.5, with only significant differences between both clones and clone 3.10.

#### 3.1.6. TNF-*α*

All MSC clones caused a very marked decrease in this cytokine, both in the absence (Table 1) and the presence of mitogen. Stimulation with PHA (Table 2) leads this cytokine to a significant increase in the absence of MSC clones. The clone 1.7 was the one with the lowest capacity to decrease levels of this cytokine and showed significant differences with the rest of clones.

#### 3.1.7. IFN-*γ*

Without MSC clones, this cytokine increased significantly after adding PHA (Table 2) to the culture. However, this increase was practically suppressed in the presence of clones 1.10, 1.22, and 3.5, which also showed significant differences with clones 1.7 and 3.10, which seemed to have no effect on IFN-*γ* synthesis and secretion. Interestingly, these last two clones were able to promote IFN-*γ* production in the absence of stimulus (Table 1).

#### 3.1.8. IL-4

Cytokine levels were nearly undetectable in the different growing conditions, reaching maximum values of 20 pg/mL. Without stimuli (Table 1) and only in the presence of clone 3.10, a slight increase in cytokine levels could be observed.

#### 3.1.9. IL-5

This cytokine was not detected in the absence of stimulus (Table 1). However, stimulation of PBMC with PHA (Table 2) resulted in an increase around 170 pg/mL in all cases, which was significantly lower in the presence of MSC clones. Significant differences were found between clones 1.10 and 3.10, which respectively caused the greatest and the least reduction.

#### 3.1.10. IL-9

This cytokine was only detected in some conditions under PHA stimulation (Table 2), reaching peak levels of around 500 pg/mL, which was significantly lower in the presence of different clones of MSC.

#### 3.1.11. IL-10

There was a clear trend towards an increase in this cytokine when PBMC were cocultured with MSC clones without stimuli (Table 1). PHA produced an increase in this cytokine (Table 2), which was lower with MSC clones vs. PBMC alone, except for the case of clone 1.7. This decrease was significant for clones 1.10 and 3.10.

#### 3.1.12. IL-13

In the absence of stimuli (Table 1), this cytokine was only detected at very low levels in the presence of clone 1.10. PHA stimulation (Table 2) increased this cytokine in the absence of MSC clones and, in a lesser degree, in the presence of clones, and not even being produced in the case of cultures containing clones 1.10 and 3.5, which showed significant differences with clone 3.10. However, these differences were significant for all clones vs. PBMC alone.

### 3.2. Proliferation Assays

#### 3.2.1. CD3^+^ Purified Lymphocytes and MSC Cocultures

**Figure 1:**
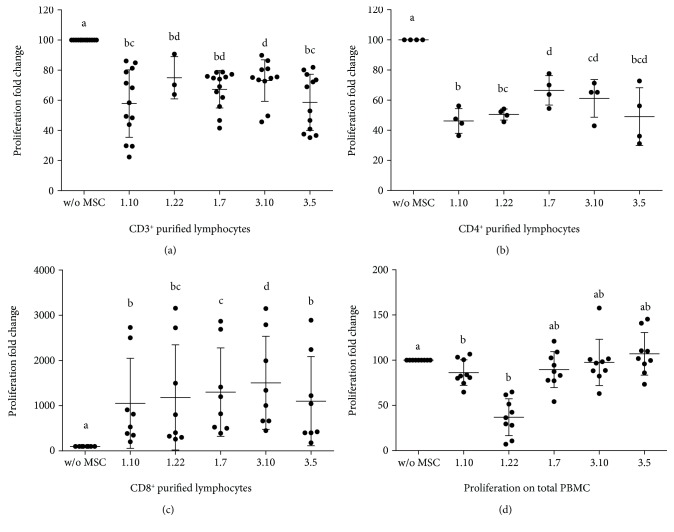
Lymphocyte proliferation in PHA-stimulated (10 *μ*g/mL) cultures in the presence or absence of different mesenchymal stem cell (MSC) clones. Ratio: MSC : lymphocytes (1 : 10). (a) Coculture of MSC-CD3^+^ purified cells (*n* ≥ 3). (b) Coculture of MSC-CD4^+^ purified cells (*n* = 4). (c) Coculture of MSC-CD8^+^ purified cells (*n* = 8). (d) Culture of PMBC and conditioned media of different MSC clones (*n* = 9). Results expressed as median (SD). *p* values obtained by ANOVA (LSD test). Means containing equal letters are not significant to each other (*p* < 0.05).

MSC clones did not induce proliferation of CD3^+^ T lymphocytes in the absence of stimuli (data not shown). In the presence of PHA, MSC clones promoted a strong inhibition of proliferation, clones 1.10 and 3.5 being the most suppressive ones (Figure 1(a)). Interclonal analysis showed small significant differences of clones 1.10 and 3.5 vs. clone 3.10.

#### 3.2.2. CD4^+^ Purified Lymphocytes and MSC Cocultures

**Figure 2:**
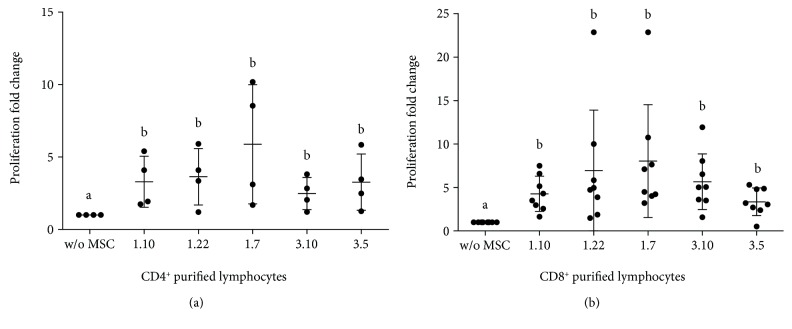
Lymphocyte proliferation nonstimulated cultures in the presence or absence of different mesenchymal stem cell (MSC) clones. Ratio: MSC : lymphocytes (1 : 10). (a) Coculture of MSC-CD4^+^ purified cells (*n* = 4). (b) Coculture of MSC-CD8^+^ purified cells (*n* = 8). Results expressed as median (SD). *p* values obtained by ANOVA (LSD test). Means containing equal letters are not significant to each other (*p* < 0.05).

Under no stimulation conditions, a significant increase in proliferation of purified CD4^+^ lymphocytes was detected in the presence of all MSC clones vs. control (Figure 2(a)) but not between clones. However, in the presence of stimulus, cocultures showed a significant decrease in proliferation (Figure 1(b)), being clones 1.7 and 1.10 the least and the most inhibitors, respectively. Finally, interclonal analysis showed significant differences of clone 1.7 vs. clones 1.10 and 1.22. The same than before, differences were also found between clones 1.10 and 3.10.

#### 3.2.3. CD8^+^ Purified Lymphocytes and MSC Cocultures

**Figure 3:**
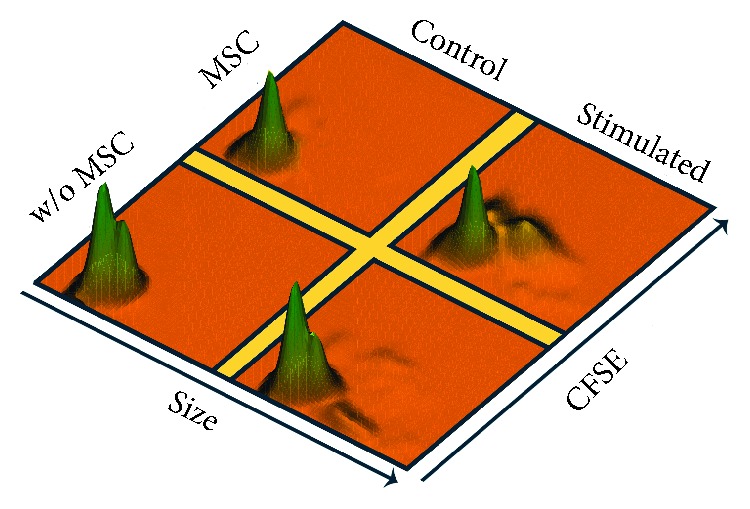
3D histograms of MSC-CD8^+^ lymphocyte cocultures, showing proliferation versus cell size. Ratio: MSC : lymphocytes (1 : 10). The height of the histogram is proportional to the number of cells. Top, without stimulus and with MSC; left, without stimulus and without MSC; right, with stimulus and with MSC; bottom, with stimulus and without MSC. Histogram representative of one of nine cocultures.

**Figure 4:**
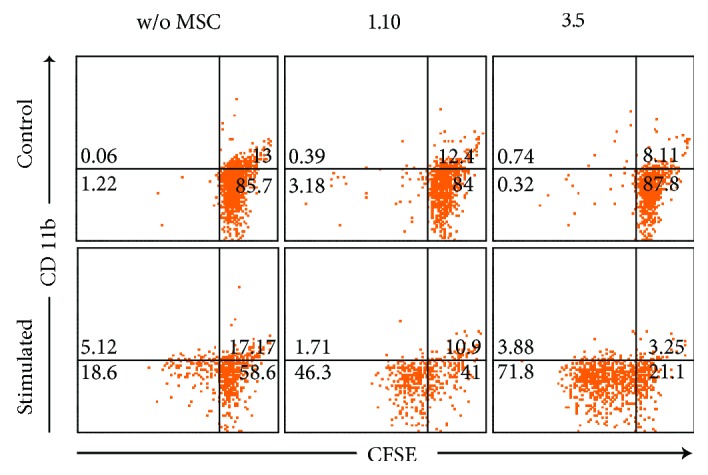
Dot-plots of one representative MSC-CD8^+^ lymphocyte cocultures, showing expression of CD11b vs. CFSE on CD8^+^ cells.

The presence of MSC clones produced a significant increase in CD8^+^ lymphocyte proliferation (Figure 2(b)). Interclonal analysis showed values close to statistical significance between clones 1.10–1.7, 1.10–3.10, 1.7–3.5, and 3.10–3.5. In the absence of MSC, PHA caused a slight increase in the size of part of the purified CD8^+^ lymphocytes (Figure 3). However, MSC caused a clear increase in the proliferation of this population (Figure 1(c)), showing variable degrees of significance for clones 1.10 (*p* = 0.0304), 1.22 (*p* = 0.0339), 1.7 (*p* = 0.0105), 3.10 (*p* = 0.0062), and 3.5 (*p* = 0.0241). Interclonal analysis showed that clone 3.10 promoted the highest level of proliferation when compared to the rest of clones. Clone 1.7 also showed differences with clones 1.10 and 3.5, resulting in an intermediate level of proliferation. Integrin CD11b was analysed for proliferated CD8^+^ lymphocytes in cocultures with clones 1.10 and 3.5, resulting in a clear majority of CD11b^−^ population (Figure 4).

#### 3.2.4. PBMC and MSC-CM Cultures

In the absence of stimulus, no significant differences were found in lymphocytes' proliferation between the different MSC-CM and control. In the presence of PHA (Figure 1(d)), all CM slightly decreased lymphocyte proliferation vs. control, reaching significant differences for clones 1.10 (*p* = 0.0198) and 1.22 (*p* < 0.0001). Interclonal analysis did not show significant differences.

### 3.3. Treg Analysis

**Figure 5:**
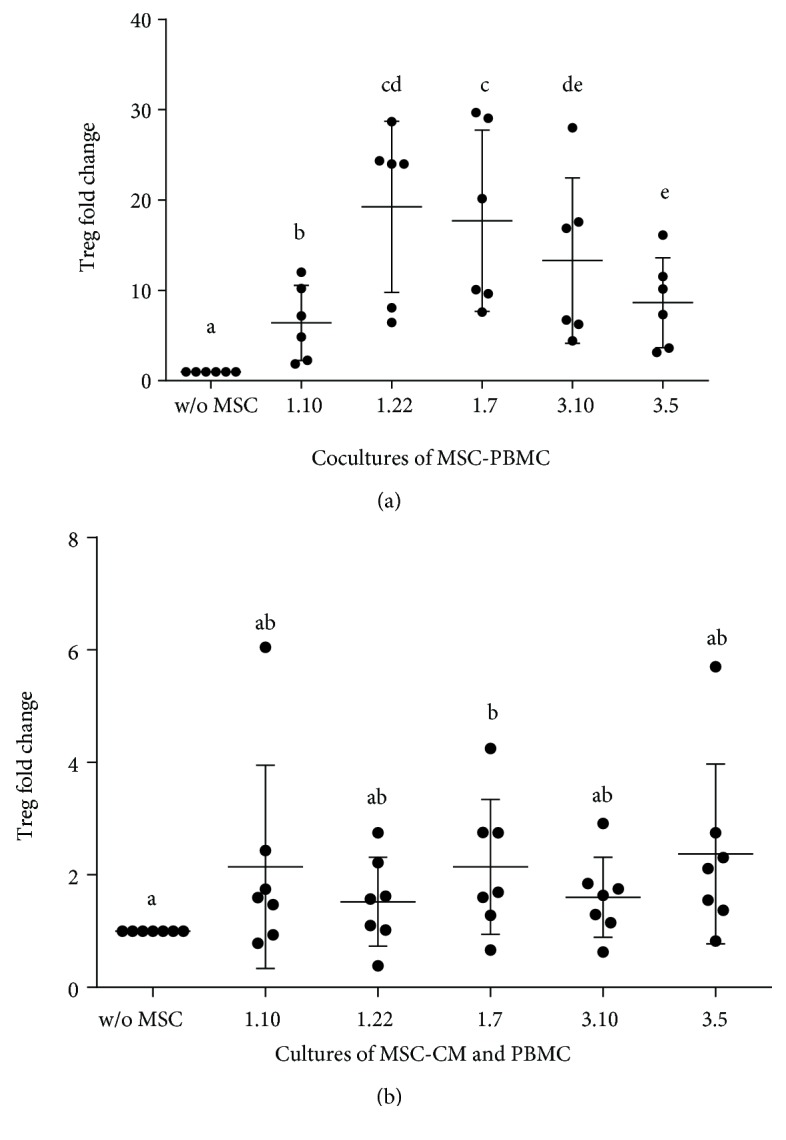
Fold change of Treg cells. (a) In cocultures of PBMC and different MSC clones. Ratio: MSC : PBMCs (1 : 10) (*n* = 6). (b) In cultures of PBMC supplemented with conditioned media from different MSC clones (*n* = 8). Results expressed as median (SD). *p* values obtained by ANOVA (LSD test) (*p* < 0.05). Means containing equal letters are not significant to each other (*p* < 0.05).

The fold change of Treg cells (CD4^+^CD25^+^FoxP3^+^) increased when PBMC were cocultured with MSC clones [1.10 (*p* = 0.0247), 1.22 (*p* = 0.0052), 1.7 (*p* = 0.0095), 3.10 (*p* = 0.0216), and 3.5 (*p* = 0.0131)] (Figure 5(a)). Numerous differences were observed in the interclonal comparison, being clones 1.22 and 1.7 which promoted the highest changes.

All the CM also caused an increase in the expression of Treg (Figure 5(b)), although significant differences were only found in the case of clone 1.7-CM. However, the *p* values for the rest of CM vs. control indicate a strong tendency to increase Treg (between 0.0635 and 0.1444). No interclonal differences were found.

### 3.4. Viability Assay

**Figure 6:**
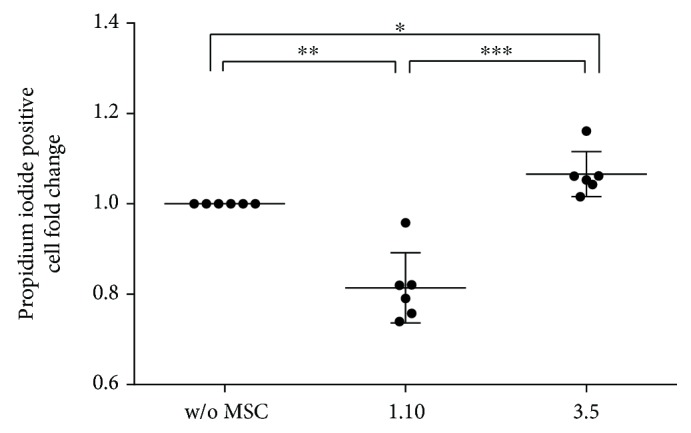
Fold change of propidium iodide (IP) positive cells in UV-stimulated cultures of PBMCs pretreated with clones 1.10 and 3.5. Ratio: MSC : PBMCs (1 : 20) (*n* = 6). Results expressed as median (SD). *p* values obtained by ANOVA (LSD test). ^∗^*p* ≤ 0.05, ^∗∗^*p* ≤ 0.01, and ^∗∗∗^*p* ≤ 0.001.

Viability analysis of PBMC showed significant differences between UV light treatment and negative control for all growing conditions. In the absence of UV light, the clones did not exert any influence on the viability of PBMC. However, in UV-treated cells, the presence of clone 1.10 significantly decreased PBMC mortality (*p* = 0.0020), contrary to clone 3.5, which slightly increased it (*p* = 0.0225) (Figure 6), resulting in a significant difference between the two clones (*p* = 0.0006).

### 3.5. Comparative Analysis of MSC Clones

**Figure 7:**
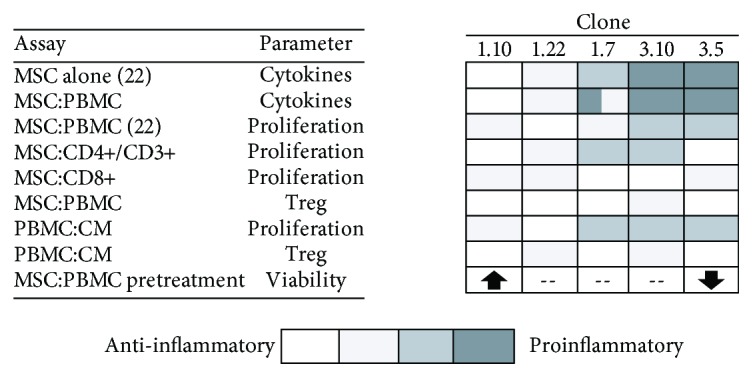
Qualitative comparison of the different factors analysed in the different MSC clones, showing a wide range of profiles, from anti-inflammatory (white) to proinflammatory (black), which identifies the individual properties of each clone.

The set of data obtained in this study shows that the different MSC clones present a wide range of behaviours that goes from anti-inflammatory to proinflammatory. This allows us to characterize the different clones according to different factors, such as cytokine profile, inhibition of different lymphocyte populations, modulation of Treg cells, and the effect on viability (Figure 7).

## 4. Discussion

Apart from the multiple applications that MSC may have in regenerative medicine, one of the most important properties of these cells is that they are able to interact with different cell populations of the immune system. Although the mechanisms by which MSC exert immunomodulation are not yet fully characterized, we know that they are highly heterogeneous [25].

When MSC are cultured as colonies derived from a single cell, each clone shows a variable degree of plasticity [26]. This could be related to the different isolation protocols, the growing conditions and/or the number of passages, because certain clones with proliferative advantages could replace the rest. In addition, some studies have shown that the source of the MSC also compromises their differentiation towards one or more lineages [26, 27]. According to our previous studies, the clones analysed in this work present heterogeneity in their differentiation potential towards osteogenic and adipogenic lineages [23] and also in their membrane phenotype, cytokine profiles, and in their capacity to inhibit PBMC's proliferation [22].

In the present study, when clones of MSC are in coculture with PBMC, increases and decreases in both proinflammatory (Th1) and anti-inflammatory (Th2) cytokines were also detected. Without stimulation, all cocultures showed a significant increase of IL-1*β* and IL-10 and a significant decrease of TNF-*α*. However, IL-1*β* increased between 3 and 5 times and IL-10 between 65 and 75 times, depending on the clone, thus supporting the well-known immunosuppressive character of MSC. Clone 1.7 is related to the greater increase of IL-1*β* and the smaller decrease of TNF-*α*, opposite to clone 1.22. Regarding the rest of the cytokines, IL-2 and IL-4 were not detected in the presence of clones 1.10, 1.7, and 3.5. Only in the presence of clone 1.10, IL-22 and IL-13 were detected, while IFN-*γ* was only present for clones 1.7 and 3.10. PHA caused a general increase of the different cytokines, although in a different way to that observed in the absence of stimuli. All clones promoted an increase of IL-17A, except for clone 1.10. In the case of TNF-*α*, all clones behaved the same than in the absence of stimuli, although more intensely. All clones also resulted in a decrease of anti-inflammatory cytokines, being clones 1.7 and 3.10 the ones promoting the lowest inhibition. At the same time, these clones were related with the highest levels of several proinflammatory cytokines such as IL-1*β*, IL-22, and IFN-*γ*. Taking into account all the aforementioned data, they could be considered as the most proinflammatory clones. For the rest of the clones, the opposite was true. As a summary of the cytokines analysis, clones of different individuals may behave similarly, while clones of the same individual may behave differently. Theoretically, we could find in every person a wide range of clones with slightly different profiles, ranging from a proinflammatory to an immunosuppressive phenotype.

MSC can inhibit proliferation of cell populations of the immune system, without being inherently immunogenic [28], supporting their allogenic and even xenogeneic, therapeutic use. Results are, however, incongruent [29] and might lie in the MSC heterogeneity [27]. In this study, we have analysed the immunomodulatory capacity of MSC clones to inhibit the proliferation of lymphocyte subpopulations. MSC clones did not induce cell proliferation of cocultured unstimulated purified CD3^+^ T lymphocytes. However, a slight proliferation was detected in cocultures with purified CD4^+^ and CD8^+^ lymphocytes. This fact would probably be conditioned by the final cytokine environments, resulting from the different interactions between leukocyte populations present in the culture in every case. Under stimulatory conditions, clones 1.10 and 3.5 were two times more effective suppressors than the other clones. PHA stimulated cocultures showed an increase in purified CD8^+^ lymphocyte proliferation. As far as we know, this is the first study reflecting this amazing behaviour, as it is contrary to what has been previously described in the literature [30], although it should be noted that to our knowledge, there are very few studies that have determined the effect of MSC on this purified lymphocyte population, through proliferation assays. It has to be remarked that PHA stimulation promoted a very low level of proliferation without MSC. However, the observed effects on cell size would indicate that certain activation occurred but being insufficient to translate into proliferation. This could be determined by the absence of factors derived from other leukocyte populations, such as Th cells, present in the rest of the analysed cocultures. By adding MSC to the culture, clones could provide or promote secretion of these deficient factors. This is consistent with other authors, which argue that inhibition of cell proliferation would be mainly determined by its stimulating effect on Treg lymphocytes [30, 31]. Interestingly, again different patterns between clones are observed. In fact, clones 1.7 and 3.10 behave similarly to each other, exhibiting the highest degree of proliferation of CD8^+^ lymphocytes. The opposite was true for clones 1.10, 1.22, and 3.5. We decided to measure the degree of expression of the CD11b membrane antigen on CD8^+^ proliferating cells. The proliferating population was essentially CD11b^−^, having these CD8^+^CD11b^−^ lymphocytes been associated with immunosuppressive properties [32], indicating again that MSC would mainly be exerting their effect by affecting a regulatory subpopulation, as previously mentioned for Treg cells. Finally, we have also analysed the effect of CM obtained from the different clones on PBMC proliferation inhibition, being lower than the one observed under the presence of clones and only reaching a significant value for clone 1.22.

In general terms, the above-mentioned effects on lymphocyte proliferation inhibition would confirm once again the differences between clones when exerting their immunomodulatory abilities therefore showing how important could be a previous characterization of these clones when using cell-therapy protocols. The different effects observed on Tc lymphocytes proliferation would further justify this characterization.

We also analysed the effect of MSC clones and its CM on Treg population. Clone 1.22 caused the highest increase of Treg, showing this clone and its CM the greatest capacity to inhibit lymphocyte proliferation as well as high levels of IL-2, this latter needed for Treg development [33]. However, IL-2 was not detected for clones 1.10 and 3.5, thus explaining the lower observed Treg number. Nevertheless, clone 1.7 promoted similar Treg fold changes than clone 1.22 but did not promote IL-2 secretion. This fact could be determined by the presence of other soluble factors such as TGF-*β*, also important for Treg development (not measured in this study). Interestingly, fold change of FoxP3 induced by clones reproduces the same pattern as the one obtained for the analysis of IL-10, this latter being a characteristic cytokine of Treg. These results seem to be again in line with studies suggesting that inhibition promoted by MSC would be mainly mediated through an indirect effect on Treg cells. CM produced the same effect as MSC but with a lower intensity. Only clone 1.7 showed a statistically significant effect on the Treg's exchange rate, reflecting again variability between clones. This is consistent with our data obtained on lymphocyte proliferation, and although support that MSC in the culture seem to be necessary to get maximum immunomodulatory effects. CM could also be an option that moreover would avoid the additional complications often happening when using cells for therapy.

Several studies have determined the capacity of MSC and their CM to increase cell viability rescuing apoptotic cells [32, 33] and to induce apoptosis in tumour cells, encouraging fact for cell therapy [34]. In this study, we have analysed the effect of clones 1.10 and 3.5 on the viability of PBMC. Clone 1.10 was able to significantly reduce cell mortality, while clone 3.5 had just the opposite effect. Given that UV is an apoptotic activator, it is reasonable to think that our clones would be modifying apoptotic routes. So far, most of the results of the current study showed similar behaviours for all clones, differing only in their intensity. However, these latest results show that heterogeneity of MSC could be also favouring antagonistic responses. These facts could be causing unknown unpredictable side effects on the viability of other cell populations involved in the pathology being treated and might influence the final clinical results.

In addition, when the effect of clones was analysed from the perspective of differences between individuals (by grouping clones 1 and 3 separately), we did not find significant differences between them (data not shown). This absence of differences supports the possibility of getting a wide range of clones with distinct behaviours from each patient that could be used in therapy according to the particular needings in each case. However, when using heterogeneous MSCs populations, these specific behaviours of clones could be masked.

## 5. Conclusions

In summary, all the aforementioned results show how important can be a previous characterization of MSC for cell therapy protocols. This characterization should be approached from different perspectives, not only by looking for the ability of these cells to differentiate into different tissues, but also by analysing their immunomodulatory abilities that would result from the combination of the multiple effects exerted by these cells. Cell therapy protocols using a clone or a combination of clones rather than the usual heterogeneous populations would facilitate their reproducibility and therefore would allow a better prediction of the pursued effects, also preventing the usual problems derived from the cellular heterogeneity applied to different individuals. From our point of view, many of the therapies that are not obtaining the expected results could have different outcomes if their design was approached from the perspective of the clones or their CM, depending on whether they wanted to obtain a longer or more specific effect, according to what was observed in our ex vivo results and also in concordance with what was described by other authors [35]. In fact, we could design ex vivo the most appropriate personalized therapy in each case, depending not only on the pathology to be treated, the duration of the desired effect, and its degree of activity but also on the individual who will receive it. According to our results, this design could be done based on the different capacities of these clones to activate or inhibit a given leukocyte population, to promote a certain balance of anti- or proinflammatory cytokines or a specific Th17/Treg balance, or even to modify the viability of specific cellular populations. As a conclusion, the heterogeneity of MSC, far from being a handicap for cell therapy, could be used to get more optimized designed protocols by using clones instead of whole cell populations.

## Figures and Tables

**Table 1:** Cytokine concentrations (pg/mL) in unstimulated cocultures of MSC clones with PBMCs (mean ± SD).

	Concentration of cytokines (pg/mL) in PBMC : MSC cocultures					
	w/o MSC	1.10	1.22	1.7	3.10	3.5
IL-1*β*	253.6 (248.7)^a^	996.2 (434.1)^bc^	788.0 (490.8)^b^	1133.6 (689.8)^c^	1071.4 (440.5)^bc^	919.4 (425.1)^bc^
TNF-*α*	106.4 (72.5)^a^	14.6 (12.8)^b^	7.3 (6.7)^b^	29.0 (9.5)^ab^	18.4 (10.4)^b^	5.5 (9.6)^b^
IL-10	2.1 (3.6)	129.8 (35.3)	148.6 (76.8)	150.7 (109.7)	128.6 (95.1)	161.7 (89.0)
IFN-*γ*	3.9 (6.7)	—	—	45.7 (79.1)	59.5 (72.5)	—
IL-4	3.6 (6.2)	—	3.6 (6.2)	—	7.2 (12.4)	—
IL-2	30.6 (52.9)	—	32.9 (57.0)	—	16.9 (29.2)	—
IL-22	—	18.0 (31.1)	—	—	—	—
IL-13	—	10.9 (18.9)	—	—	—	—
IL-17A	—	—	—	—	3.8 (6.6)	—
IL-9	1.0 (1.7)	—	—	—	—	—
IL-5	—	—	—	—	—	—
IL-12p70	—	—	—	—	—	—

Groups (*n* = 3) containing the same letters are not statistically different for ANOVA (LSD), *p* < 0.05.

**Table 2:** Cytokine concentrations (pg/mL) in phytohemagglutinin-stimulated (10 *μ*g/mL) cocultures of MSC clones with PBMCs (mean ± SD).

	Concentration of cytokines (pg/mL) in PBMC : MSC cocultures					
	w/o MSC	1.10	1.22	1.7	3.10	3.5
IL-1*β*	2055.1 (151.5)^a^	2344.6 (247.9)^a^	2025.1 (498.3)^a^	2809.0 (662.8)^b^	2770.9 (712.0)^b^	2136.7 (740.2)^a^
TNF-*α*	416.8 (23.5)^a^	32.4 (7.2)^c^	42.5 (26.6)^c^	208.6 (101.8)^b^	79.9 (29.7)^c^	14.1 (24.4)^c^
IL-10	819.4 (433.2)^a^	352.2 (62.4)^b^	531.8 (190.3)^bc^	783.1 (312.2)^ac^	455.0 (45.3)^b^	531.4 (148.0)^bc^
IFN-*γ*	2512.4 (1829.8)^a^	359.9 (252.7)^b^	270.3 (207.2)^b^	3180.4 (1441.5)^a^	2093.2 (1854.4)^a^	186.1 (177.3)^b^
IL-4	1.9 (3.3)	—	—	—	—	—
IL-2	27.8 (31.6)	18.9 (32.8)	18.9 (32.8)	22.5 (39.0)	—	—
IL-22	51.53 (77.31)^ab^	—^a^	12.5 (21.7)^ab^	40.7 (70.5)^ab^	81.3 (71.0)^b^	—^a^
IL-13	998.0 (246.7)^a^	—^b^	110.7 (96.7)^bc^	123.2 (108.4)^bc^	210.6 (325.7)^c^	—^b^
IL-17A	549.3 (330.5)^ab^	350.1 (90.4)^a^	936.2 (508.1)^cd^	906.6 (613.8)^cd^	723.3 (307.0)^bc^	1017.5 (403.0)^d^
IL-9	527.3 (126.4)^a^	—^b^	20.2 (20.6)^b^	119.3 (117.2)^c^	45.7 (73.9)^bc^	2.9 (2.6)^b^
IL-5	168.2 (120.8)^a^	10.3 (17.9)^b^	58.5 (50.8)^bc^	63.9 (50.4)^bc^	83.4 (110.2)^c^	31.4 (54.4)^bc^
IL-12p70	—	—	—	37.4 (22.8)	—	—

Groups (*n* = 3) containing the same letters are not statistically different for ANOVA (LSD), *p* < 0.05.

## Data Availability

The GraphPad Prism files and all the LMD cytometry data used to support the findings of this study are available from the corresponding author upon request.
